# Correction: A graph neural network-based approach for predicting SARS-CoV-2–human protein interactions from multiview data

**DOI:** 10.1371/journal.pone.0339211

**Published:** 2025-12-16

**Authors:** Sumanta Ray, Syed Alberuni, Alexander Schönhuth

There are errors in Fig 1. The figure is displayed incorrectly in Panel A and Panel B. Please see the correct Fig 1 here.

**Fig 1:**
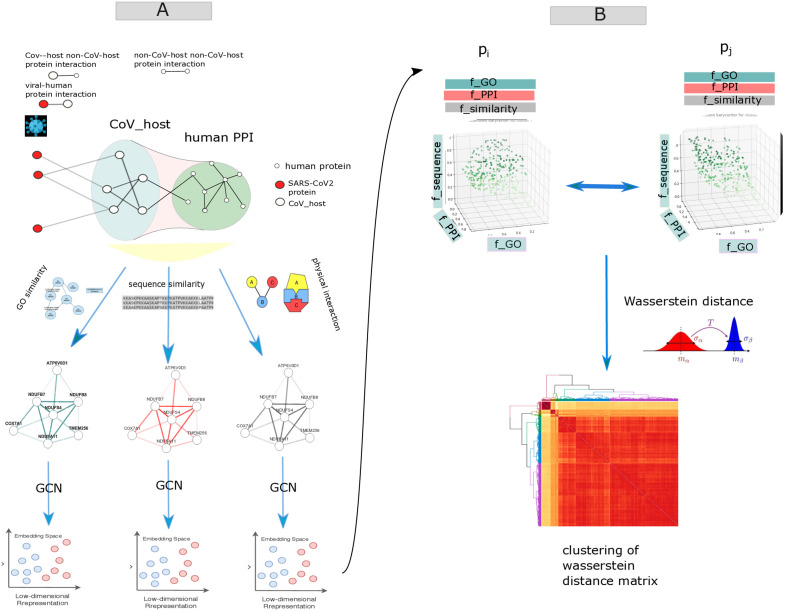
The analysis pipeline begins by constructing three distinct interaction networks (panel. A): physical protein-protein interactions (PPI), gene ontology (GO)-based functional similarity, and protein sequence similarity networks.

Each network is separately encoded into embeddings using Graph Convolutional Networks (GCNs). Subsequently, embeddings from each network are integrated to represent each protein within a three-dimensional unit cube, where each dimension corresponds to a distinct biological perspective (panel B). Protein-protein similarity is computed using the Wasserstein distance, capturing the minimum “cost” to align multivariate distributions of protein embeddings derived from the three networks (panel C). Hierarchical clustering is applied to group similar proteins into clusters based on the Wasserstein distance matrix. Finally, probable targets or host factors of SARS-CoV-2 are predicted by identifying non-CoV-host proteins clustered closely with experimentally validated CoV-host proteins.
