# Supplementary material for: A graph neural network-based approach for predicting SARS-CoV-2–human protein interactions from multiview data
Source: PLoS One. 2025 Sep 25;20(9):e0332794. doi: 10.1371/journal.pone.0332794 (PMC12463271; doi:10.1371/journal.pone.0332794)
Supplement: S2 Table — (PDF) [file pone.0332794.s002.pdf]

Supplementary Table-2: GO enrichment analysis of the top 100 predicted human proteins: Top 10 significantly enriched GO biological process terms. Gene ratio is the proportion of input genes in the term; background ratio is the term's representation in the background.

GO Term	Description	Gene Ratio	Bg Ratio	p-value	FDR/q-value	Top Contributing Genes
GO:0046718	Viral entry into host cell	8/100	52/20000	3.1×10^{-6}	1.5×10^{-4}	ACE2, TMPRSS2, CTSL, FURIN, NRP1, CD209, HSPA5, ANPEP
GO:0050776	Regulation of immune response	12/100	250/20000	1.2×10^{-5}	5.6×10^{-4}	TNF, NFKB1, STAT1, IFNAR1, IL6R, OAS1, DDX58, IRF3, CXCL10, PML, MX1, IFITM3
GO:0072657	Protein localization to membrane	10/100	180/20000	4.6×10^{-4}	1.7×10^{-3}	SLC2A1, ATP1A1, VAPA, GOLGA7, RAB7A, CD46, SCARB1, TMEM41B, SNX27, VPS35
GO:0097190	Apoptotic signaling pathway	7/100	90/20000	2.7×10^{-3}	7.8×10^{-3}	BAX, BCL2, CASP3, CASP8, FAS, DDIT3, TP53
GO:0035456	Response to interferon-beta	6/100	64/20000	4.2×10^{-3}	1.1×10^{-2}	IFIT1, IFIT2, IFIT3, ISG15, MX1, OAS2
GO:0009615	Response to virus	9/100	200/20000	8.5×10^{-3}	2.0×10^{-2}	OAS1, DDX58, IFITM3, IRF3, MX1, IFIT1, ISG15, BST2, PML
GO:0016032	Viral process	7/100	101/20000	1.1×10^{-2}	2.4×10^{-2}	ACE2, FURIN, HSPA5, ANPEP, NRP1, CD209, SCARB1
GO:0034340	Response to type I interferon	5/100	80/20000	1.9×10^{-2}	4.3×10^{-2}	ISG15, MX1, OAS2, IFITM3, IFIT3
GO:0051607	Defense response to virus	6/100	110/20000	2.5×10^{-2}	5.2×10^{-2}	OAS1, IFIT1, IFIT2, IFIT3, DDX58, IRF3
GO:0006955	Immune response	8/100	340/20000	2.7×10^{-2}	5.7×10^{-2}	TNF, NFKB1, STAT1, IFNAR1, IL6R, OAS1, DDX58, IRF3
