# Supplementary material for: A graph neural network-based approach for predicting SARS-CoV-2–human protein interactions from multiview data
Source: PLoS One. 2025 Sep 25;20(9):e0332794. doi: 10.1371/journal.pone.0332794 (PMC12463271; doi:10.1371/journal.pone.0332794)
Supplement: S1 Fig — The rows of the sub-matrices represent the CoV-host proteins interacting with a particular SARS-CoV-2 protein, and the columns represent the non-CoV-host proteins inferred by those CoV-hosts from the predicted list. (PDF) [file pone.0332794.s003.pdf]

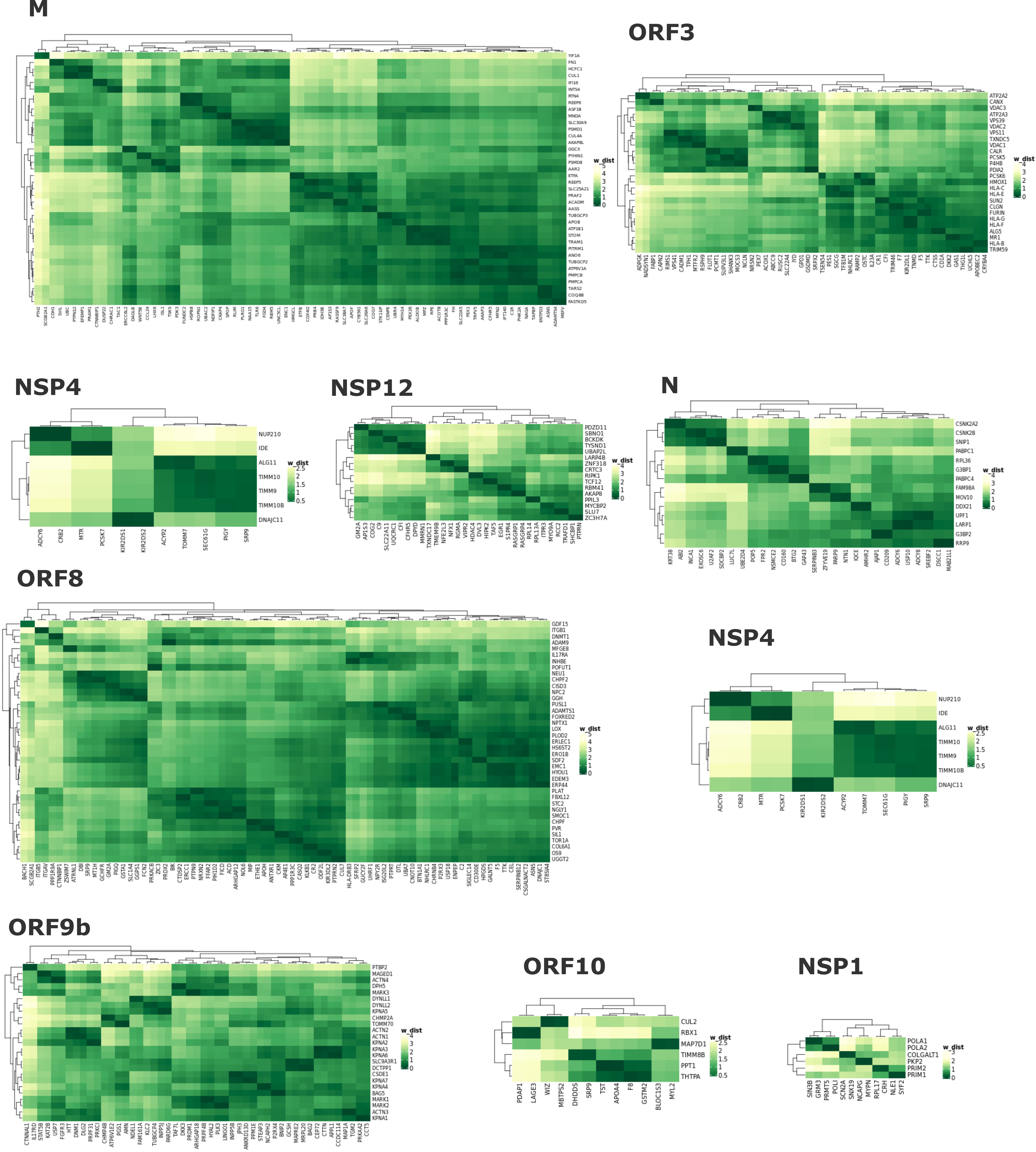
M
ORF3
NSP4
NSP12
N
ORF8
NSP4
ORF9b
ORF10
NSP1
w_dist
NUP210
IDE
ALG11
TIMM10
TIMM9
TIMM10B
DNAJC11
ADCY6
CRB2
MTR
PCSK7
KIR2DS1
KIR2DS2
ACYP2
TOMM7
SEC61G
PIGY
SRP9
CUL2
RBX1
MAP7D1
TIMM8B
PPT1
THTPA
PDAP1
LAGE3
WIZ
MBTPS2
DHDDS
SRP9
TST
APOA4
F8
GSTM2
BLOC1S3
MYL2
POLA1
POLA2
COLGALT1
PKP2
PRIM2
PRIM1
